# Phase 1 clinical study of cell therapy with effective-mononuclear cells (E-MNC) for radiogenic xerostomia (first-in-human study) (FIH study on E-MNC therapy for radiogenic xerostomia)

**DOI:** 10.1097/MD.0000000000020788

**Published:** 2020-06-26

**Authors:** Yoshinori Sumita, Naoki Iwamoto, Makoto Seki, Takako Yoshida, Ryo Honma, Mayumi Iwatake, Seigo Ohba, I. Takashi, Yuka Hotokezaka, Hiroshi Harada, Shinichiro Kuroshima, Kazuhiro Nagai, Takayuki Asahara, I Atsushi Kawakam, Izumi Asahina

**Affiliations:** a Basic and Translational Research Center for Hard Tissue Disease; b Department of Immunology and Rheumatology, Division of Advanced Preventive Medical Sciences, Nagasaki University Graduate School of Biomedical Sciences, Nagasaki; c CellAxia Inc., Tokyo; d Department of Regenerative Oral Surgery, Unit of Translational Medicine; e Department of Radiology and Cancer Biology, Nagasaki University Graduate School of Biomedical Sciences; f Transfusion and Cell Therapy Unit, Nagasaki University; g Department of Applied Prosthodontics, Nagasaki University Graduate School of Biomedical Sciences, Nagasaki; h Department of Regenerative Medicine Science, Tokai University School of Medicine, Isehara, Japan.

**Keywords:** cell-based therapy, macrophage, peripheral blood mononuclear cells, xerostomia

## Abstract

**Background:**

Treatment for most patients with head and neck cancers includes ionizing radiation with or without chemotherapy. This treatment causes irreversible damage to salivary glands in the irradiation field accompanied by a loss of fluid-secreting acinar cells and a considerable decrease of saliva secretion. There is currently no adequate conventional treatment for this condition. In recent years, we developed an effective culture method to enhance the anti-inflammatory and vasculogenic phenotypes of peripheral blood mononuclear cells (PBMNCs), and such effectively conditioned PBMNC (E-MNC) therapy has shown promising improvements to the function of radiation-injured salivary glands in preclinical studies. However, the safety and effect of E-NMC therapy have yet assessed in human. The objective of this ongoing first-in-man study is to assess the safety, tolerability, and in part the efficacy of E-MNC therapy for treating radiation-induced xerostomia.

**Methods/design:**

This phase 1 first-in-man study is an open-label, single-center, two-step dose escalation study. A total of 6 patients, who had no recurrence of head and neck cancer over 5 years following radiation therapy and suffered from radiation-induced xerostomia, will receive a transplantation of E-NMCs derived from autologous PBMNCs to a submandibular gland. The duration of the intervention will be 1 year. To analyze the recovery of salivary secretion, a gum test will be performed. To analyze the recovery of atrophic salivary glands, computed tomography (CT), and magnetic resonance imaging (MRI) of salivary glands will be conducted. The primary endpoint is the safety of the protocol. The secondary endpoints are the changes from baseline in whole saliva secretion and salivary gland atrophy.

**Discussion:**

This will be the first clinical study of regenerative therapy using E-MNCs for patients with severe radiation-induced xerostomia. The results of this study are expected to contribute to developing the low-invasive cell-based therapy for radiation-induced xerostomia.

**Trial registration:**

This study was registered with the Japan Registry of Clinical Trials (http://jrct.niph.go.jp) as jRCTb070190057.

## 1 Introduction

Treatment with radiotherapy either alone or in combination with surgery and/or chemotherapy for head and neck cancers causes a loss of fluid-secreting acinar cells, and this adverse effect gives rise to irreversible damage to salivary gland function in 63% to 93% of patients.^[1]^ Therefore, such patients usually suffer considerable morbidity, because dysfunction of salivary glands leads to severe xerostomia (dry mouth).^[1,2]^ Xerostomia frequently causes severe dental caries, aggravation of periodontal disease, oropharyngeal infections, and diminished mucosal wound healing.^[3]^ These symptoms increase the incidence of dysphagia and severely impact patient quality of life.^[4]^ Recently, to reduce radiation damage to salivary glands, approaches such as brachytherapy, intensity-modulated radiation therapy (IMRT), and heavy particle beam therapy have shown merit, but a significant proportion of patients still experience salivary gland atrophy and hypofunction.^[5]^

Current pharmacological approaches for radiation-induced xerostomia, including sialagogues or saliva substitutes, aim to stimulate saliva secretion from the residual acinar cells or provide alternatives that mimic natural saliva. However, these approaches are usually not satisfactory, because their effectiveness is only temporary.^[6]^ Furthermore, although sialagogues such as pilocarpine hydrochloride, a cholinergic agonist, have been frequently used to relieve radiation-induced xerostomia, they include major limitations resulting from common side effects such as sweating and gastrointestinal upset.^[6,7]^ Thus, a significant improvement of these symptoms in patients has yet to be observed using current pharmacological approaches. Therefore, developing adequate treatments for the restoration of radiation-induced atrophic salivary glands is urgently required.

We recently developed a novel culture method for peripheral-blood mononuclear cells (PBMNCs) with serum-free medium containing 5 recombinant growth factors (stem cell factor, thrombopoietin, vascular endothelial growth factor, interleukin-6, and Flt-3 ligand), and found that this culture method can successfully enhance the anti-inflammatory and vasculogenic potential of PBMNCs in a short period (5–7 days).^[8]^ The resulting effectively conditioned PBMNCs (E-MNCs) contain an enriched population of definitive CD11b/CD206-positive (M2 macrophage-like) cells. When E-MNCs were injected intra-glandularly into a mouse model of radiation-induced atrophic salivary glands, salivary secretory function gradually recovered after 4 weeks post-irradiation (post-IR), and was 4-fold higher than that of non-transplanted model mice at 12 weeks post-IR. Enhanced green fluorescent protein (EGFP)-expressing E-MNCs were detected in a portion of vascular endothelium and perivascular gland tissues at 2 weeks post-IR, but only in microvessel walls at 3 weeks. Between 4 and 12 weeks post-IR, mRNA-expression and histological analyses revealed that E-MNC transplantation reduced the expression of inflammatory genes and increased the level of tissue regenerative activities such stem cell markers (c-Kit and Sca-1), cell proliferation, and blood vessel formation. At 12 weeks post-IR, the areas of acinar and ductal cells regenerated, and the glands had less fibrosis. Therefore, this effective conditioning of PBMNCs is a simple, rapid, and efficient method that provides a noninvasive source of therapeutic cells for regenerating radiation-injured atrophic salivary glands.

The objective of this phase 1 first-in-man study has been to assess the safety and in part the efficacy of an intra-glandular injection of autologous E-MNCs for treating radiation-induced atrophic salivary glands and xerostomia. In this article, we provide the detailed design of this clinical study, which is ongoing and is registered with the Japan Registry of Clinical Trials.

## 2 Methods/design

### 2.1 Study design

**Figure 1:**
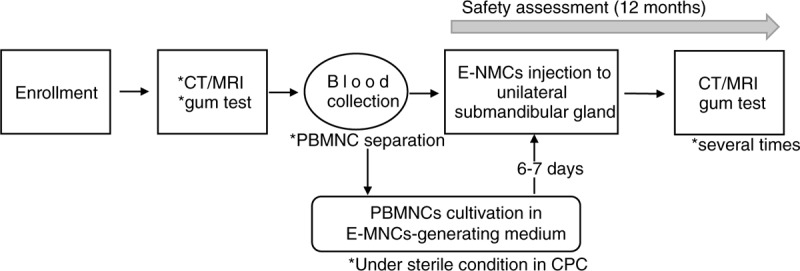
Study design. E-MNCs are generated in a hematopoietic stem cell expansion medium containing recombinant human SCF, recombinant human Flt-3 ligand, recombinant human TPO, recombinant human VEGF, and recombinant human IL-6. CT = computed tomography, MRI *=* magnetic resonance imaging, PBMNCs *=* peripheral blood mononuclear cells, E-MNC *=* effective mononuclear cell, CPC *=* cell-processing center.

This study is a phase 1 first-in-man clinical trial to assess the safety, tolerability, and efficacy of E-MNC therapy for radiation-induced xerostomia. This study will be conducted at a single center (Nagasaki University Hospital). A total of 6 patients with radiation-induced xerostomia will receive transplantation of E-MNCs. A two-step protocol will be used: the first 3 patients will be classified as “step 1” and will each receive transplantation with a low dose (8 × 10^6^) of E-MNCs. The other 3 patients will be classified as the “step 2” group and will each receive transplantation with a high dose (2 × 10^7^) of E-MNCs. The duration of the study is 1 year. The study design is summarized in Figure 1.

This study was approved by Kyushu University Certified Special Committee for Regenerative Medicine (NA8150001) and was registered with the Japan Registry of Clinical Trials (http://jrct.niph.go.jp) as jRCTb070190057. We will conduct this study in accordance with the principles of the Declaration of Helsinki and the Japan Good Clinical Practice guidelines and in compliance with the Ethical Guides for Medical Studies in Human Subjects (promulgated on December 22, 2014) and the Act on the Protection of Personal Information and related regulatory notifications.

### 2.2 Inclusion criteria

Patients must meet all of the following requirements to be considered for entry in the study:

1. xerostomia after radiotherapy for head and neck cancer;
2. it has been 5 years after that radiotherapy, with no recurrence of head and neck cancer (complete remission);
3. the complaint of dry mouth and a gum test result of <10 ml/10 minute;
4. atrophy of salivary gland confirmed by CT or MRI;
5. has undergone scaling, received tooth brushing guidance, and has maintained good oral hygiene;
6. age 20 to 75 years;
7. written informed consent (IC) can be obtained from the patient him/herself;
8. the patient has the intention and ability to visit a hospital; and
9. has the intention to use barrier contraception during the study period.

### 2.3 Exclusion criteria

The patients who participate in this study will be required to undergo blood collection for the isolation of PBMNCs. Patients who show low hemoglobin counts will thus be excluded: <12.5 g/dl for males and <12.0 g/dl for females when 200 ml blood collection is conducted, and <13.0 g/dl for males and <12.5 g/dl for females when >200 ml blood collection is conducted. The other major exclusion criteria are as follows:

1. xerostomia caused by a salivary gland tumor;
2. any other malignancy or sepsis;
3. severe autoimmune or endocrinological disease;
  coagulation abnormality (PT < 50% or outside the APTT range 23.5–42.5 seconds);
4. positivity of syphilis test/HBV antigen/HCV antigen/anti-HTLV-1 antibody/anti-HIV antibody;
5. liver dysfunction (higher or lower value of 2 biomarkers of liver function than the following criteria: AST 10–40 IU/L, ALT 5–45 IU/L);
6. pregnancy;
7. risk of allergy regarding the drug used in this study;
8. allergy regarding penicillin G, amphotericin B, or streptomycin;
9. transmissible spongiform encephalopathy;
10. dementia;
11. a psychiatric disorder such as depression;
12. a smoking habit;
13. being judged by the clinical investigator as an inappropriate patient for this study.

### 2.4 Outcome measurements

**Figure 2:**
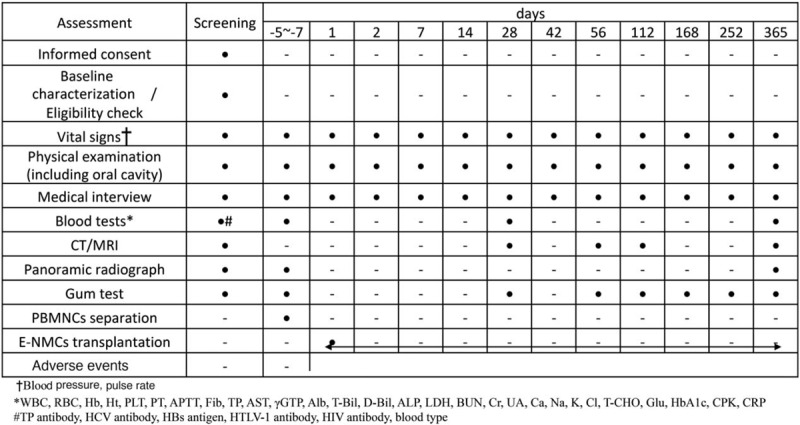
Treatment schedule and outcome measures. CT = computed tomography, MRI *=* magnetic resonance imaging, PBMNCs = peripheral blood mononuclear cells, E-MNC = effective mononuclear cell.

Study visits will take place at baseline (approx. 1 month before the surgery) and on specified days after the surgery. The assessments are presented in Figure 2. CT and MRI of salivary glands will be performed at baseline and after 28, 56, 112, and 365 days. Using these imaging methods, the size of atrophic glands and the volume of atrophic tissues will be calculated. Panoramic radiographs will be taken at baseline and after 365 days. A physical examination including the oral cavity and a safety assessment will be performed at every visit. Gum tests to evaluate the quantities of salivary secretion at 14, 28, 56, 112, 168, 252, and 365 days are planned.

The primary endpoint is the safety/tolerability of the protocol. The safety assessment, which conducted by independent safety monitoring board, will be done by a two-step process. The first 3 enrolled patients will receive transplantation of a low dose of E-MNCs (8 × 10^6^), and their safety will be assessed at ≥4 weeks after the surgery. If there are no serious adverse events in any of these 3 patients, the next 3 patients enrolled will receive transplantation of a high dose of E-MNCs (2 × 10^7^), and their safety will be assessed.

The secondary endpoints are the change from baseline in salivary secretion assessed by gum tests and the change in salivary gland atrophy (i.e., the rate of atrophic parts in salivary glands, and the volume of atrophic glands) assessed based on the CT/MRI findings.

### 2.5 Adverse events (AEs)

All adverse events (AEs) that occur between the surgery and 1 year post-surgery will be recorded. If necessary, the investigators will administer treatments. A serious AE (SAE) is defined as any adverse reaction resulting in any of the following outcomes: a life-threatening condition or death; a condition that requires inpatient hospitalization or prolongation of an existing hospitalization, threatening to cause disability or death; a congenital anomaly; or a birth defect. All SAEs will be reported to the IRB and certificated committee for regenerative medicine by the responsible investigator.

### 2.6 Method for the generation of E-MNCs

The generation of E-MNCs, specific conditions of which were established by CellAxia Inc. (Tokyo, Japan), will take place under sterile conditions in a cell-processing center (CPC) at Nagasaki University Hospital. With the method to be used, 100 to 240 ml of peripheral blood is obtained and PBMNCs are separated by density gradient centrifugation with the use of a separation medium (Histopaque-1077, Sigma Aldrich, St Louis, MO). The isolated PBMNCs are seeded on 6-well Primaria tissue culture plates (BD Biosciences, San Jose, CA) at a density of 4 × 10^6^ cells/3 ml of medium/well and cultured for 6 to 7 days under the 5G-culture system as previously described.^[8]^

Briefly, PBMNCs are cultured in serum-free medium (Stemline II Hematopoietic Stem Cell Expansion Medium; Sigma Aldrich) with recombinant human stem cell factor (SCF), recombinant human Fms-related tyrosine kinase 3 (Flt-3) ligand, recombinant human thrombopoietin (TPO), recombinant human vascular endothelial growth factor (VEGF), and recombinant human interleukin-6 (IL-6) (all from Peprotech, Rocky Hill, NJ), which is modified human quality- and quantify-controlled culture system as described in our previous work.^[8,9]^ After 6 or 7 days of cultivation, the cells (effectively conditioned PBMNCs; E-MNCs) are harvested.

### 2.7 Evaluation of E-MNCs

Quality testing on cell appearance is executed at 5 days of cultivation before harvesting of E-MNCs. Then, at 6 or 7 days, harvested E-MNCs are assessed for viable cell number, viability, and the presence of the macrophage fraction via flow cytometry, and then it is decided whether or not to administer E-MNCs to patients. Quality testing for E-MNCs also includes the presence of bacteria, virus, mycoplasma, or endotoxin contamination.

### 2.8 Surgical method for the injection of E-MNCs in the submandibular gland

The surgical procedure will be performed with local anesthesia in an operating room. The sterile E-MNCs suspension (400 μl, 8 × 10^6^, or 2 × 10^7^) will be injected into a unilateral submandibular gland guided by ultrasound; 100 μl of the E-MNCs suspension will be injected in 4 areas of the submandibular gland to ensure an equal distribution of the suspension. The participants will be administered a pain reliever such as a non-steroidal anti-inflammatory drug (NSAID). Wound cleaning will be done the next day and after 1 week post-surgery.

### 2.9 Data collection and management

The personal data and the clinical data of the participants will be coded and saved separately. All data will be recorded in case report form (CRF) by appropriate and authorized persons (principal investigator or sub-investigators). All data related to personal information such as the consent form will be kept in locked cabinets. The investigator maintains a personal identification list (patient numbers with the corresponding patient names) to enable records to be identified.

The monitoring of study compliance and data collection will be done by the principal investigator and the sub-principal investigators authorized (not involved in this study) staff. During the study, the monitor will make regular site visits to review protocol compliance, assess the laboratory procedures, and ensure that the study is being conducted according to protocol requirements.

### 2.10 Trial status

The first version was published on November 3, 2017 and was last updated on March 31, 2020. Recruitment started in April 2019 and is expected to finish in 2021.

## 3 Discussion

For the restoration of radiation-induced atrophic salivary glands, experimental approaches applying cell-based therapy have recently been investigated.^[10–13]^ Among these approaches, intra-glandular or -venous injections of mesenchymal stem cells (MSCs) in an irradiation mouse model have been examined frequently and show the potential of this strategy to slow the atrophic process.^[12]^ MSCs can display paracrine effects via releasing anti-inflammatory, vasculogenic, and anti-apoptotic cytokines to damaged glands, and these effects induce the anti-inflammatory phenotypes of activated macrophages at injured sites.^[14,15]^ Such macrophages function in neo-vascularization and subsequent tissue regeneration.^[16–18]^ These experimental approaches strongly suggest that cell-based therapies are promising as an alternative remedy for atrophic salivary glands. Indeed, more recently, Grønhøj et al have used adipose tissue-derived MSC therapy in a clinical study and demonstrated efficacy in a phase 1/2 trial for radiation-induced xerostomia.^[19]^ However, the autologous cell sources reported to date such as bone marrow, adipose tissue, or dental mesenchymal tissue require invasive procedures to harvest at the donor site.^[11,20,21]^ In addition, securing a sufficient number of highly functional MSCs in culture to ensure their therapeutic effects remains a challenge, particularly for elderly patients.^[22,23]^ Therefore, as an alternative strategy, we have developed a novel culture method to obtain therapeutic cells from a readily available and low invasive source, the peripheral blood. E-MNCs may affect the restoration of atrophic tissues more directly than MSCs, because E-MNCs contain an enriched population of M2 macrophage-like cells, which have similar properties to anti-inflammatory macrophages induced by transplanted MSCs at injured sites.

This first-in-man phase 1 study aims to evaluate the safety and in part the efficacy of E-MNC therapy using an intra-glandular injection for patients who suffer from radiation-induced xerostomia. E-MNCs are obtained with a serum-free primary culture system in only 5 to 7 days after 100 to 240 ml of blood is collected, and then their suspensions can be injected directly into salivary glands without any surgical procedures. E-MNCs are considered to have low tumorigenicity, as found in pluripotent stem cells, because E-MNCs are composed of autologous blood cells such as lymphocytes, monocytes/macrophages, and their progenitors.^[8]^ Therefore, this strategy is thought to have low invasiveness and be applied easily and safely in the clinic. However, the cell processing method for E-MNCs is simple but newly developed. In addition, the transplantation of E-MNCs may have the potential risk of activating tumor growth and metastasis due to their vasculogenic properties. Therefore, we planned this clinical study as a phase 1 first-in-man two-step dose-escalation study to verify the safety of E-MNC therapy restricted to patients who have had no recurrence of head and neck cancer over 5 years following radiation therapy. As mentioned above, Grønhøj et al have used advanced adipose tissue-derived MSC therapy in a phase 1/2 trial for radiation-induced xerostomia.^[19]^ These studies will help improve the methodology for cell-based therapy and inform the design of subsequent studies on the safety and potential efficacy of this intervention.

## Acknowledgments

The authors would like to thank our colleagues and staff at the Oral Surgery Department, Nagasaki University Hospital for their support.

## Author contributions

**Conceptualization:** Yoshinori Sumita, Makoto Seki, Takayuki Asahara, Izumi Asahina.

**Data curation:** Yoshinori Sumita, Izumi Asahina.

**Formal analysis:** Yoshinori Sumita, Mayumi Iwatake, Kazuhiro Nagai, Izumi Asahina.

**Funding acquisition:** Yoshinori Sumita, Izumi Asahina.

**Investigation:** Yoshinori Sumita, Takako Yoshida, Ryo Honma, Myumi Iwatake, Seigo Ohba, Takashi I, Yuka Hotokezaka, Hiroshi Harada, Shinichiro Kuroshima.

**Methodology:** Yoshinori Sumita, Izumi Asahina.

**Project administration:** Yoshinori Sumita, Izumi Asahina

**Supervision:** Izumi Asahina, Atsushi Kawakami.

**Writing – original draft:** Yoshinori Sumita, Naoki Iwamoto.

**Writing – review & editing:** Naoki Iwamoto, Kazuhiro Nagai, Izumi Asahina
